# Chicago Medical Society; Meeting of January 3d

**Published:** 1887-03

**Authors:** 


CHICAGO MEDICAL SOCIETY:

*Stated Meeting, January 3, 1887.*—THE PRESIDENT, EDMUND J. DOERING, M.D., in the Chair.

DR. E. J. KUH read a paper on

THE ETIOLOGY AND CURE OF ASTHMA.

The work of Wilhelm Hack on the radical treatment of migraine, asthma, hay-fever and other neuroses, has received very inadequate recognition in this country. He teaches that the rhinoscope must be as indispensable an instrument for all physicians as the thermometer and stethoscope.

The value of Hack's discovery, that asthma nervosum is a reflex disease, with, usually, the nose as the starting point, can best be appreciated by one who himself, for many years, struggled against the disease. If I, therefore, in the course of this paper, class myself among my own patients, I shall do so with the view of bringing the subject within closer range.

The form of asthma of which I wish to treat exclusively is asthma nervosum, or "Essentielles Asthma" of the Germans. Some persons never get beyond a slight hint of asthma. They will from time to time make a heaving, sighing motion, or complain of præcordial fullness with or without palpitation, or of sudden drowsiness, or dream heavily at night and complain of dullness, lassitude and headache in the morning. This latter condition has many gradations, the culmination of which is nightmare. We must, therefore, learn to distinguish between an incubus of gastric and of respiratory origin. Other half-asthmatics complain only of a fleeting, leaden heaviness in the limbs amounting almost to pain; the same sensation of which so many true asthmatics complain after an asthmatic night. The typical asthma nervosum is a neurosis occurring in paroxysms.

The patient may or may not feel an aura. He will generally, towards evening, or when he lies down, or awakes in the night, begin to wheeze. This wheezing may be associated with itching in the nose, or sneezing, or coughing; the attacks last an indefinite time, and generally end with the expectoration of a transparent glassy mucus. Such patients are often free from asthma during the day. Physical and chemical irritants, such as dust, sudden changes in temperature, the inhalation of certain gases, and a long series of idiosyncrasies which we find enumerated in text-books, can induce an attack. But the recumbent position is the most uniform, exciting cause of the single paroxysms. Such patients may be free from chronic bronchitis, chronic emphysema, heart-, kidney-, intestinal- and uterine disease : hence the term "Essentielles Asthma."

It is merely an evasion to say that asthmatic paroxysms are induced by bronchial spasms, or by hyperæmia of the bronchial lining, or by the presence of Leyden's crystals, or by phrenic spasm, or by bulbar irritation, or by exudative bronchiolitis. For any one of these presumable causes would demand a first cause, in order to merit etiological dignity.

A true etiology of asthma had therefore to be discovered, and Hack did it in the following manner : He found that he could experimentally produce glottis-spasm by touching the turbinated bodies of a sensitive individual with a probe. He then reasoned as follows : A nasal mucous membrane which shows merely slight affection, and which is not deadened in its sensibility by thickening and hypertrophy, is perhaps a better surface for exciting reflexes than one which shows evident signs of disease.

He systematically examined the nose of every patient who, for whatever ailment, came within his reach. He learned to make one distinction very rapidly, namely : that what is usually termed hypertrophic nasal catarrh is a twofold condition, which produces quite opposite effects. In the anatomically true rhinitis hypertrophica the mucous membrane is really thickened, hypertrophied through chronic inflammation. Pressure with a probe meets with a certain unyielding resistance, and there is a purulent, crusty secretion. This form does *not* give rise to reflex disturbances.

But there is another form, a pseudo-hypertrophy, the importance of which it is Hack first pointed out. It is that transitory swelling of the cavernous tissue of the inferior and middle turbinated bodies, which has of late been so often described. In this form the nose may either have a very dry, itchy sensation, or show copious watery secretion. Compression with a probe gives the air-pillow reaction. Such individuals show fleeting alternate or synchronous obstruction of the nasal cavities. Often, when examining the nose of the patients, we notice sudden engorgements and collapse, so that Hack's term erectility is not an exaggeration. These cavernous bodies with their frequently anæmic covering form a link in certain morbid reflexes, and when this link is destroyed through operative intervention, the reflexes cease. No symptom is more frequently overlooked by patients than transitory nasal obstruction. Most patients will positively deny its existence, until it is demonstrated to them. Therefore the assurance of an asthmatic that his nose has always appeared healthy is of no value.

The theory of Hack is a simple one, and although it does not cover all the ground, is a very satisfactory one. He says that the turbinated bodies become engorged through various irritants, and that this vaso-dilatatory disturbance is transmitted to the bronchial tubes in asthma. The turbinated bodies act as accumulators for reflexes, store them up, as it were, and then transmit them to other parts. A destruction of the nasal swelling removes the reflexes. The experiences of numerous writers since 1883 corroborate the correctness of Hack's discovery.

By way of illustration I could not, I believe, select a better type of asthma of long standing than that of my own person. Twenty years ago, when I was 8 years of age, I became subject to so-called colds in the head and on the chest. They increased in severity and frequency from year to year, so that my surroundings were often puzzled to find an explanation for each outbreak. Presently nightly dyspnœa began to set in, in the following manner: During the day my respiration was quite free, but as soon as my head touched the pillow, the first wheeze set in; the paroxysms were very severe. They ceased, after lasting throughout the night, in the morning, with the usual expectoration of glassy mucus

During the day there was never any difficulty, except when occasioned by laughter. Laughter would infallibly cause itching under the chin and between the scapulæ, then I would cough convulsively and the attack was upon me. But the recumbent position was the surest exciting cause. During the first years I also suffered from that form of conjunctivitis which is now known to arise from nasal disorder.

The greater part of 1870 to 1875 I spent in the Swiss mountains, where I was entirely well. During this period I developed a peculiar idiosyncrasy towards dinner. In the midst of the meal I would invariably for weeks would be seized with a convulsive cough, so severe that it threw me to the ground. Asthma was never absent in these attacks. Then, at other times, one or two or three sneezes would initiate an asthmatic attack; or sometimes, especially after traveling, I would sneeze sixty or seventy times without intermission. In those years I had the sensation as if the asthma were brought on by a swelling which seemed to begin above and behind the palate (it was associated with intense itching; which I attempted to relieve by rubbing my tongue against the hard palate), and traveled downward to the posterior pharynx, then seemed to skip the larynx and continued from the trachea downward. This phenomenon lasted a few seconds, and then the attack began. Railroad travel would invariably cause a night of asthma. One hotel, at which I was frequently obliged to stop in Germany, adjoined a stable, and was regularly the cause of some of the severest attacks.

The inhalation of Kidder's asthma pastilles—the only palliative I ever used successfully—gave me very great relief. They not only immediately terminated an attack, but also prevented their occurrence for the next few hours.

As soon as I became acquainted with Hack's articles in the *Berliner klin. Wochenschrift*, of 1882, and with his monograph in 1883, I commenced stricter self-observation, and found the following:

As soon as I lay down, my nose would become obstructed. The occlusion corresponded to the side on which I lay. By turning over, the occluded side would open and the other close. To have any part of the nasal mucous membrane touched by a probe gave such intense pain that I could not suppress an outcry. I could bring on an attack of asthma by rubbing my alæ nasi against the septum.

Never did I feel the slightest dyspnœa when nasal respiration was free, and never was nasal respiration obstructed but what I felt asthmatic distress.

Under these circumstances there could be no hesitation, and I had the galvano-caustic "destruction" of both inferior turbinated swellings performed. I have found the radical obliteration of the entire inferior turbinated bodies almost an impossibility. Hack demands, and my experience confirms the correctness of his view, that the radical cure of asthma demands the radical destruction of the cavernous erection.

What is it that causes nasal occlusion? I have observed myself so closely in this regard, and have so many corroborative observations of intelligent patients, that I can make these positive statements:

Firstly, the fullness of the turbinated bodies is regularly influenced by gravitation, and corresponds to the position of the head.

It is furthermore influenced by the temperature, and probably much more so by artificial warmth than by cold; an over-heated room will almost invariably cause swelling in such patients. But the most dangerous and permanent cause of nasal obstruction is the inhalation of dust.

When are we to operate on asthmatics? The more recent the asthmatic trouble and the more pronounced the nasal symptoms, the better the prognosis. When complicated with chronic bronchitis and chronic emphysema, the outlook is generally bad. A most thorough examination of heart, lungs, kidneys and intestines should precede any operative interference. In cases of cardiac and nephritic asthma with nasal complications, I have *never* cauterized. In cases of long standing, say fifteen or twenty years, in which in the first years the nasal symptoms were very pronounced, but in later years have almost or entirely disappeared, cauterization is sometimes successful, but generally is unsuccessful.

Cases in which the asthma is more or less constant and has lost its paroxysmal nature, give a doubtful prognosis. It has been a matter of experience with me, that these patients to whom the inhalation of Kidder's pastilles, or the application of cocaine to the nose (four per cent. solution on cotton), gives relief, afford a much better prognosis than others.

In asthmatics in which coughing precedes the attack and all nasal symptoms are missing, nasal cauterization will cure, if the cough is a so-called nasal cough.

There are a number of asthmatics, fortunately a minority, who seemingly offer a good prognosis, but with whom, for unknown reasons, the operation will fail. There can now be no doubt that there are other starting points for reflexs in the respiratory tract, besides the nose. The works of Trautmann and Tornwaldt have already added the vault of the pharynx to this list.

The bronchial tubes themselves can act as a starting point, as I can demonstrate on myself when I walk against a piercing wind, or inhale vapors of sulphurous acid with my nose plugged. So that, as Hack himself warningly says, we must not over estimate the applicability of his discovery.

We must accuse the nose *per exclusionem.* Examine every patient thoroughly in every direction, and examine the nose *last*, is what I should like to advise.

About the operation itself, little is to be said. It is, as far as we know. absolutely harmless. I have performed many hundred cauterizations without any noteworthy complications. I have never had any traumatic infection. I insufflate iodoform or iodol upon the wound, introduce a pledget of cotton for a few days, and keep my instruments aseptic.

The results are, on the whole, extremely gratifying. Asthma of many years' standing is sometimes broken after the very first cauterization. Almost all patients are relieved and many cured in the strict sense of the word. Some have relapses, which additional cauterization will remove. Others again may relapse with a new reflex sensitiveness in other parts.

PROFESSOR J. A. ROBISON, in opening the discussion, said: The facts, which are indeed facts, that have been related in this paper are of interest not only to the specialist but to the general practitioner. It has been a fact long known to specialists that obstruction of the passage of air through the nares will give rise to asthma, and a great number of articles have been written on this subject. It has also been demonstrated that when operations have been performed that cleared away these obstructions the relief from the asthmatic attacks was complete. This can be easily demonstrated by any physician. Cases of nasal polypus are quite frequent, and they do not always fall under the care of a specialist. The operation is generally a very simple one; almost any physician, without special training, can remove nasal polypi, and it is really wonderful to find how many cases of asthma are thus cured. As to asthma being due to other causes, he had no doubt of the truth of the observation made by the author, that is, that transitory swelling which takes place in the turbinated bodies in cases of mild irritation. He presumed we have all noticed that when we are affected with an acute coryza and go to bed at night the narium of the side on which we lie becomes obstructed, and if we turn over the other side will become obstructed. This is undoubtedly due to the force of gravitation in a great many cases where the mucous membrane is especially sensitive. There is no doubt that in a great many cases by the irritation of a probe, or the inhalation of dust, coughing can be produced resulting in asthmatic attacks.

Therefore this demonstrates that reflex irritation of the nares is one of the causes of asthma, and it points out very clearly the method of treatment which should be instituted. The author has rendered a service in showing that there are such a large number of cases in which by destroying the turbinated bodies we can prevent the occurrence of reflex asthma. It would have been an interesting question to solve whether, in the case of the author's special experience, a respirator worn over the nose so that the air could not pass through the nose unless filtered, would have been of any benefit in preventing the recurrence of the asthma.

DR. H. MARTYN SCUDDER: About five years ago, when practicing in India, where he had to ride on horseback a great deal in the sun and breathe a great deal of dust, he suffered frequently from acute attacks of coryza, accompanied occasionally by bronchitis and slight asthma. The nose was not much obstructed, and when an attack of coryza came on, fifteen minutes' sleep would often cause it to pass away. Gradually the attacks became more severe and were accompanied and followed by some obstruction. When in London more than three years ago, Doctor Mackenzie wanted to cauterize his nose, but it was before the days of cocaine, and he decidedly objected, as he thought the remedy worse than the disease. Since coming to Chicago he has been troubled less than when abroad. Quite recently he had his nose cauterized by Dr. E. Fletcher Ingals, and it has certainly relieved the trouble to a very great extent. His experience, however, was somewhat different from Doctor Kuth's, as the cauterization gave considerable trouble for a week or two. It was followed by soreness and even by slight chills, and it made him feel out of sorts for about a fortnight, but it was successful in relieving the obstruction, and he has had no more asthma or bronchitis, although once in a while he still suffers from attacks of coryza.

DR. JOSEF ZEISLER said: Professor Schnitzler, of Vienna, has published a number of cases in which decidedly polypus of the nose has caused asthma, and where by the removal of the polypus the asthma was cured. He can confirm what Doctor Kuh has said in regard to the effectiveness of the galvano-cautery. He had a case of a boy 12 years old, who had nearly all his lifetime had chronic eczema of the hands and asthma. Believing that the asthma was the casual relation to the eczema he referred the patient to Doctor Kuh for treatment of the former trouble, while he prescribed local applications for the hands. Very soon both affections were cured and have remained so for the last year.

DR. H. N. MOYER asked what the author means by the term essential asthma, whether he means reflex asthma or something different?

DOCTOR KUH, in closing the discussion, said: "By essential asthma he of course means, as he had been attempting to explain all the evening, reflex asthma; the same asthma which text-books classify as idiopathic or nervous or essential asthma. In lieu of these clouded expressions we have now, fortunately, a term by which we express an etiological meaning, namely, nasal asthma. It teaches us again, that the term neurosis always smacks of the hypothetical; and that when we speak of any pathological condition as a neurosis, we do so in order to cover ignorance. An asthmatic individual is not necessarily a "nervous" one, although he, of course, is not blind to the fact that some unknown factor must come into play in order to affect disease through nasal reflex. In regard to Doctor Zeisler's remarks on the connection between polypi and asthma, he did not know that Schnitzler, of Vienna, had published forty cases of nasal polypus with asthma. He was greatly surprised that such a publication should have escaped his notice. He quoted Michel, of Berlin, as having reported 135 cases of polypus without asthma. There is no doubt that sometimes nasal polypi cause asthma, but as far as he is aware, only exceptionally so. Hack found that when a patient had polypus with asthma and he left the polypus untouched and cauterized only the turbinated bodies, the asthma disappeared, although the polypi remained in the nose. He thinks there can be no better evidence of the relative innocence of polypi than that experiment. He has been asked whether, if he had worn a respirator he would have been free from asthma in traveling. He found that to be the case. For when he plugged his nose with cotton while traveling he remained free from asthma. Doctor Scudder said that nasal cauterization gave him trouble for weeks. This could only have been through wound complication. An asthmatic may have very severe trouble for a week or less after cauterization, on account of the eschar.

In order to show how careful one must be in diagnosis he would like to interpolate the following description: A patient with the mildest form of asthma, namely, the occasional involuntary deep, sighing inspiration, consulted him. The examination was negative with the exception of slight tympanites (the abdomen should always be carefully examined in such cases) and slight swelling of the inferior turbinated bodies. He treated his mild constipation for weeks without any benefit to his respiratory trouble. Then he cauterized, also without effect. At last he discovered that his alæ nasi were so pliable that when he inhaled through the nose they collapsed and occluded the nares. In regard to the claim that injections of boracic acid solution into the nose will relieve asthma, he would simply refer to the uniformly condemnatory verdict of all specialistic practitioners against the use of the nasal douche in such cases.

PROFESSOR ALBERT WING, Pathologist to Cook County Hospital, showed

A HEART WITH ATHEROMA AT THE BASE OF THE AORTA AND IN THE MITRAL VALVE, and a condition described by the Germans as *prior chronic endocarditis*. The last mentioned lesion manifests itself in the distribution of grayish streaks or patches on the endocardial surface, lying irregularly distributed over it. When this lesion has proceeded far enough fatty degeneration follows, shown by patches which appear slightly yellowish to the eye.

The patches upon the valves are upon the anterior segment of the mitral. They are simply interesting and would cause no symptoms whatever. He did not know that such a case has any further interest than that these things very frequently exist, and in his experience more than a majority of cases upon which autopsies are held at the County Hospital, present lesions of prior chronic endocarditis.

PROFESSOR WING also exhibited

A LUNG ILLUSTRATING ONE OF THE POINTS OF DIFFERENTIAL DIAGNOSIS BETWEEN A CAVITY RESULTING FROM TUBERCULOSIS AND ONE RESULTING SIMPLY FROM DILATATION OF A BRONCHIAL TUBE IN BRONCHIECTASIS.

That point is the persistence of bands, or stumps of bands, of the more resisting tissues which remain, sometimes passing across the cavity. As the fibrous tissues are more resistant than the others in the lung they are last to disappear in the necrotic process. In this specimen there are a few cavities in the apex, some of them large, and the tubercular infiltration extends entirely to the base of the lower lobe of the right lung. There was extensive adhesion of the two layers of the pleura over the lung.

PROFESSOR W. T. BELFIELD asked for a repetition of the diagnosis distinction between cavities due to tuberculosis and bronchiectasis.

PROFESSOR WING said a cavity resulting from bronchiectasis has a smooth membrane, and upon washing it, no stumps of these bands can be seen upon its floor; but in a cavity resulting from tuberculosis there are always some of these stumps or bands present. Sometimes they are very short, at other times long, and at times, as in this case, they are easily seen and demonstrated.

DR. A. V. PARK read a report of

A CASE OF ANTE-PARTUM HÆMORRHAGE AT TERM; RECOVERY.

On August 5 the author was called to see Mrs. S., a well-built and intelligent American-born Irish woman. This was her ninth confinement, and she had had six miscarriages. She had received no injuries while carrying this child except running against an obstruction in the yard which gave her a slight shock. The evening previous she had had severe hemorrhage but no real labor pains. A careful examination was made and the bedding found wet with blood, but no evidences of continued uterine hemorrhage. Vaginal examination revealed a rigid undilated os high in the pelvis. At 3 o'clock examination was again made and the os found soft and the head presenting. The pains were irregular and had no effect upon the cervix. At 11 o'clock true labor pains came on; os soft and dilated, head at the brim of the pelvis, cervix rigid with each pain. The liquor amnii having all escaped with the so-called hemorrhage the expulsive efforts accomplished little. At 2:30 A.M. everything seemed favorable for an early termination of labor, but it was soon noticed that while the pains were severe they were not propulsive. The patient was restless and thirsty, and the danger of concealed ante-partum hemorrhage was realized. The only thing to be done was to deliver at once. The patient was placed across the bed and the membranes ruptured, which was followed by a small gush of blood. Then the forceps were applied and a still-born child delivered which had probably been dead six hours. The child was given to the nurse, and with the left hand over the fundus of the uterus a gentle pressure was made. The uterus soon began to contract and expel its contents, the blood and blood-clots that were forced out filling a wash-basin. The placenta was high up and normally situated, and easily removed, when the hemorrhage ceased. Ergot was given and the patient made as comfortable as possible. The author concludes that the hæmorrhage was caused by a partial separation of the normally situated placenta, and that the head of the child acted like a ball valve, which prevented the escape of the blood externally; that a portion of the blood found its way into the anniotic cavity, which would account for the slight hæmorrhage that followed the mechanical rupture of the membranes previous to delivery. He thought that in cases of internal hæmorrhage during labor the treatment should depend upon the stage of the labor and the amount of blood lost. If the patient be in danger of sinking and the os dilatable, but the head within the uterus, delivery should be performed by turning. If the loss is moderate wait until the head descends into the cavity of the pelvis. In all cases where possible forceps should be used for immediate delivery. The child is lost in almost every case of extreme hæmorrhage.

DR. A. V. [illegible]ARK reported

A CASE OF PYELITIS OF NINETEEN YEARS' DURATION, CAUSED BY A RENAL CALCULUS. RECOVERY.

The patient was 30 years old, of slight physique and nervous temperament. His sufferings were excruciating. First attack occurred when he was 11 years old, during convalescence from scarlet fever. He was an engineer, and when exposed while covered with perspiration his old trouble would inevitably follow. He had received treatment from some of the best physicians and surgeons, and had taken almost every kind of medication, but without relief. He had taken such quantities of narcotics that it required a phenomenal dose to affect him. He had never passed calculi with his urine so far as he knew, and the entire amount had been saved and examined time and again always with negative results. Morphia was given hypodermically and a careful examination made, the urine being subjected to an analysis. Then came the question, what should be done for the patient? The best surgical authorities agree that renal calculi are generally composed of uric acid or oxalate of lime. Dr. Belfield advises the injection of large quantities of alkaline water, and says that in pyelitis caused by renal culculi it affords the only hope for radical cure by medical means. Calculi have been dissolved by copious injections of simple rain-water, and the same result can be obtained by water which contains the proper ingredients to give it an alkaline reaction. On May 13 the patient was in great suffering. Morphia was administered, and a line of treatment mapped out which was followed faithfully. The patient was directed to drink Waukesha water often in large quantities, and tincture of digitalis ½ oz., fluid ex. hydrangea 2 oz., calisayæ enough to make 4 oz. were prescribed, one teaspoonful every six hours. A milk diet and ½ dr. Carlsbad sprudel salts in a glass of water before breakfast was advised. In order to facilitate the washing out process the system was relaxed by anodynes, and hypodermic injections of morphia and atropia were given and hot poultices applied. This treatment, with the injection of large quantities of water, was continued during the night. At 8 o'clock A. M. the patient was free from pain and had passed a large quantity of dark colored urine in which was a calculus weighing 14 grains, oval in shape, with numerous headlike elevations composed of uric acid. On analysis the calculus was found to be composed principally of oxalate of lime. The patient said that he could distinctly feel the stone when it dropped into the bladder The treatment was continued and the kidney troubles soon ceased. It has now been seventeen months since the last attack, and he is robust and strong.

